# Up‐regulation of ZFAS1 indicates dismal prognosis for cholangiocarcinoma and promotes proliferation and metastasis by modulating USF1 via miR‐296‐5p

**DOI:** 10.1111/jcmm.14698

**Published:** 2019-09-29

**Authors:** Zhenglong Li, Xingming Jiang, Lining Huang, Jinglin Li, Daolin Ji, Yi Xu, Kaiming Leng, Yunfu Cui

**Affiliations:** ^1^ Department of Hepatopancreatobiliary Surgery The Second Affiliated Hospital of Harbin Medical University Harbin China

**Keywords:** cholangiocarcinoma, lncRNA ZFAS1, metastasis, proliferation, USF1

## Abstract

LncRNAs has been demonstrated to modulate neoplastic development by modulating downstream miRNAs and functional genes. In this study, we aimed to detect the interaction among lncRNA ZFAS1 miR‐296‐5p and USF1. We explored the proliferation, migration and invasion of cholangiocarcinoma. The differentially expressed ZFAS1 was discovered in both tissues and cell lines by qRT‐PCR. The targeting relationship between miR‐296‐5p and ZFAS1 or USF1 was validated by dual‐luciferase assay. The impact of ZFAS1 on CCA cell proliferation was observed by CCK‐8 assay. The protein expression of USF1 was determined by Western blot. The effects of ZFAS1, miR‐296‐5p and USF1 on tumour growth were further confirmed using xenograft model. LncRNA ZFAS1 expression was relatively up‐regulated in tumour tissues and cells while miR‐296‐5p was significantly down‐regulated. Knockdown of ZFAS1 significantly suppressed tumour proliferation, migration, invasion and USF1 expression. Overexpressed miR‐296‐5p suppressed cell proliferation and metastasis. Knockdown of USF1 inhibited cell proliferation and metastasis and xenograft tumour growth. In conclusion, ZFAS1 might promote cholangiocarcinoma proliferation and metastasis by modulating USF1 via miR‐296‐5p.

## 1 INTRODUCTION

Cholangiocarcinoma (CCA) is an aggressive epithelial carcinoma originates from the biliary ducts and the second most common malignancy of primary hepatobiliary tumours with an increasing morbidity worldwide.^1^, ^2^, ^3^, ^4^ For instance, the incident of CCA reaches a number of approximately 7500 each year in the USA.^5^ The pathogenesis of CCA consists of primary sclerosing cholangitis, hepatolithiasis, biliary tract deformity, clonorchiasis and many others via massive signalling cascade alterations and molecule dysregulations, including non‐coding RNAs (ncRNAs).^6^, ^7^, ^8^, ^9^ Unfortunately, cholangiocarcinoma is still identified as a disease without effective therapeutic approaches due to the lack of effective diagnostic and prognostic biomarkers, and surgical treatments make just limited contribution to the cure of CCA.^10^, ^11^, ^12^, ^13^ Therefore, novel biomarkers and therapeutic means are imperative and tremendously needed for CCA patients.

Long non‐coding RNAs (lncRNAs), identified as a wide category of transcripts that lack the potential of coding protein, are multifunctional members of the ncRNA family, which are greater than 200 nucleotides in length.^14^, ^15^, ^16^, ^17^ Despite lacking protein‐coding potential, they are also associated with the progression of different types of cancers. Rapid advances of tumour genomics have highlighted the various function modes of lncRNAs in diverse human cancers, including CCA. For example, up‐regulated lncRNA HOTAIR promotes glioblastoma cell cycle progression, HULC cooperates with MALAT1 to aggravate liver cancer stem cells growth, and overexpressed PANDAR contributes to the promotion of cholangiocarcinoma tumorigenesis.^18^, ^19^, ^20^ Furthermore, emerging evidences have demonstrated that lncRNAs play crucial roles in the regulation of different biological processes, including regulation of mRNA transcription and post‐transcription, alternative splicing modulation, oncogenesis promotion and many others.^21^, ^22^, ^23^ Given to those evidences and oncogenic roles of variety, lncRNAs have been revealed to have the significant functions and a close relation with the progression of cancers.

Zinc finger antisense 1 (ZFAS1), located at chromosome 20, is antisense to the 5´end of ZNFX1 promoter.^24^ Askarian‐Amiri ME et al^25^ discovered that ZFAS1 was host to three C/D box snoRNAs and highly regulated in the developing mouse mammary gland. Furthermore, knockdown of lncRNA ZFAS1 could inhibit breast cancer cell proliferation and metabolic activity, which indicated lncRNA ZFAS1 a tumour suppressor. However, the following studies dramatically indicated its distinct role of cancer promotion, which refuted the tumour suppressor role of ZFAS1. For instance, lncRNA ZFAS1 is overexpressed in hepatocellular carcinoma and acts as oncogene by binding miR‐150, ZFAS1 promotes gastric cancer cell growth by repressing KLF2 and NKD2 expression, ZFAS1 is an unfavourable prognostic factor for glioma patients and promotes tumour cell malignant progression by the activation of Notch signalling pathway, and many others.^24^, ^26^, ^27^ Up to date, ZFAS1 has been demonstrated dysregulated and involved in several different cancers functions as oncogene; however, the possible biological role and prognostic capacity of ZFAS1 in CCA are still ambiguous.

In the present study, we investigated the pattern of lncRNA ZFAS1 in cholangiocarcinoma and evaluated the correlations between ZFAS1 expression, clinicopathological parameters and overall survival of CCA patients. Furthermore, we also explored the function of ZFAS1 in CCA cell proliferation, migration and invasion both in vitro and in vivo. Additionally, the effect of dysregulation of ZFAS1 on epithelial‐mesenchymal transition (EMT) and the proliferation‐related proteins were further detected.

## 2 MATERIALS AND METHODS

### 2.1 Patients and tissue specimens

Sixty‐four paired cholangiocarcinoma tissues were obtained intraoperatively from patients who underwent surgery operation at the Second Affiliated Hospital of Harbin Medical University between February 2011 and March 2013, and patients recruited had never received any chemotherapy or radiotherapy preoperatively or postoperatively. The diagnosis of cholangiocarcinoma was histopathologically confirmed, and all tissue samples were snap‐frozen in liquid nitrogen immediately and stored at −80°C until RNA extraction. The research was in compliance with the protocols approved by the Ethical Committee of the Second Affiliated Hospital of Harbin Medical University, and all experiments were undertaken with the consent of each subject.

### 2.2 Cell culture and transfection

Cholangiocarcinoma cell lines (CCLP‐1, RBE, QBC939 and HuCCT1) and normal control cell line HIBEC were preserved in the laboratory as previously described.^28^ Cells were cultured in 10% foetal bovine serum (FBS) loaded RPMI‐1640 medium (Invitrogen Life Technologies) and 100 μg/mL penicillin/streptomycin supplemented at 37°C with 5% CO_2_ in a humidified incubator. Small interfering RNAs targeting ZFAS1 (si‐ZFAS1‐1 and si‐ZFAS1‐2), USF1 (si‐USF1), negative controls (si‐ZFAS1‐NC, si‐USF1‐NC) were purchased from Shanghai GenePharma company, and so did miR‐296‐5p inhibitor and corresponding negative control (inh‐miR‐296‐5p, inh‐NC). Lipofectamine 2000 (Thermo Fisher Scientific, Inc) was used for transfection according to the manufacturer's instructions. The transfected cells were harvested 48 hours after transfection.

### 2.3 RNA extraction and quantitative real‐time PCR

Total RNA was extracted from tissues or cells using TRIzol reagent (Invitrogen). The cDNA was synthesized using a reverse transcriptase kit in accordance with the manufacturer's recommendations. The RNA quality and concentration were detected using NanoDrop 1000 Spectrophotometer (Thermo Fisher Scientific, Inc). Complementary DNA (cDNA) was reversely transcribed from RNA using a reverse transcriptase kit (Roche). The quantitative real‐time PCR (qRT‐PCR) was performed using the FastStart Universal SYBR Green Master (Roche, Germany) on a BIO‐RAD C1000 Thermal Cycler and U6, and GAPDH was used as an internal control. The 2^−ΔΔ^ *^C^* ^t^ method was performed to compute the relative expression levels. The qRT‐PCR for primers is shown in Table 1.

**Table 1:** The primers collection

Gene name	Forward primer	Reverse primer
ZFAS1	5′‐ACGTGCAGACATCTACAACCT‐3′	5′‐TACTTCCAACACCCGCAT‐3′
USF1	5′‐CACTAAACTCTGGGGCTTGTCC‐3′	5′‐CACCAGCCACTGCTAAACATCC‐3′
miR‐296‐5p	5′‐GTATCCAGTGCAGG GTCCGA‐3′	5′‐CGACGAGGGCCCCCCCT‐3′
GAPDH	5′‐GGGAGCCAAAAGGGTCAT‐3′	5′‐GAGTCCTTCCACGATACCAA‐3′
U6	5′‐CTCGCTTCGGCAGCACA‐3′	5′‐AACGCTTCACGAA TTTGCGT‐3′

### 2.4 Lentivirus production and plasmid construction

The lentiviral vector containing ZFAS1 DNA sequence (ZFAS1‐wt), the lentiviral vector containing mutated (predicted miR‐296‐5p binding sites) ZFAS1 DNA sequence (ZFAS1‐mut), the lentiviral vector containing ZFAS1 shRNA (shZFAS1) and the negative control were purchased from Shanghai Genechem company. To generate pmirGLO‐ZFAS1‐wt and pmirGLO‐USF1‐wt, ZFAS1 cDNA and USF1 3′UTR were amplified by PCR and then subcloned into pmirGLO plasmid (Generay Biotechnology). The pmirGLO‐ZFAS1‐mut and pmirGLO‐USF1‐mut were generated by the site‐directed mutagenesis kit (Stratagene). The constructs were validated by DNA sequencing.

### 2.5 Cell viability and colony formation assays

Cell counting kit‐8 (CCK‐8) assays were performed to evaluate cell viability. The transfected cells were seeded into 96‐well plates, and the proliferation of cells was evaluated every 24 hours following the manufacturer's instructions of CCK‐8 kit (Dojindo Laboratories). To quantify the absorbance of each well, a microplate reader (Tecan) was used. To detect the clonogenic capacity, colony formation assay was carried out. The transfected cells were seeded into 35 mm culture dishes and cultivated with RPMI‐1640 containing 10% FBS for 14 days until visible colonies formed. Cell colonies were fixed with paraformaldehyde before stained with 0.1% crystal violet (Beyotime) for 15 minutes and then conducted photograph and colony counting.

### 2.6 Wound healing and transwell assays

Cell migration was firstly measured by wound healing assays. Pipette tubes were used to make a scraped, acellular area. After 0 hours and 36 hours, photographs were taken to evaluate the motility of each transfected group. For transwell assay, the transfected cells were suspended in serum‐free medium and plated into the transwell chambers with a pore size of 8 µm. Cell invasion was evaluated performing the Chamber matrigel invasion 24‐well units (Costar). The assays were performed according to the manufacturer's instructions. Briefly, cells from each group were suspended in serum‐free medium and were seeded into the upper chamber. The lower chamber was filled with medium containing 10% FBS. After incubation for 24 hours, the migrated/ invaded cells in the lower chamber (below the filter surface) were fixed in 4% paraformaldehyde, stained with crystal violet solution and counted under a microscope.

### 2.7 Hoechst 33342 staining

Cells were seeded on sterile cover glasses placed in the four chambered slide. When they grew to approximately 70% confluence, cells were washed twice in ice‐cold PBS. After washing, the cells were fixed with 4% paraformaldehyde in PBS for 30 minutes at 4℃, washed twice with PBS and stained with Hoechst 33342 (Thermo Fisher Scientific) at a final concentration of 10 mg/mL at room temperature for 5 minutes. Nuclear morphology was then examined using a fluorescent microscope.

### 2.8 Target prediction and Dual‐Luciferase reporter assay

The miR‐296‐5p binding motifs in ZFAS1 and USF1 3′UTR was predicted with the help of computer‐aided algorithms: StarBase (http://starbase.sysu.edu.cn) and TargetScan database (http://www.targetscan.org), respectively. To investigate whether miR‐296‐5p directly targeted ZFAS1 and USF1 3′UTR, the reporter plasmid of wild‐type ZFAS1, reporter plasmid of mutated‐type ZFAS1, wild‐type USF1 3′UTR reporter plasmid and mutated‐type USF1 3′UTR reporter plasmid were constructed with pmirGLO‐promoter vector (Generay Biotechnology). All vectors were verified by sequencing, and luciferase activities were assessed using the Dual‐Luciferase Assay System (Promega).

### 2.9 Tumour xenograft study

The tumour xenograft formation and evaluation were reviewed and approved by the Animal Care and Use Committee of Harbin Medical University, and animals were treated with euthanasia if inevitable. CCLP‐1 cells were transfected with shZFAS1 or negative control. About 4 × 106 tumour cells were subcutaneously injected into either side of each female BALB/c nude mouse (6 weeks of age) after transfection for 48h. Tumour growth measurement was performed every 72 hours from the sixth day. The calculation for tumour volumes, euthanization, mice weight measurement and xenografts RNA evaluation were conducted as previously described.^28^

### 2.10 Protein extraction and western blot analysis

Total proteins were extracted from the cells with RIPA buffer and quantified by a BCA kit (Beyotime Biotechnology). About 50μg of extracted proteins were separated by SDS‐PAGE and then transferred onto PVDF membranes (Merck Millipore). After soaking with 5% non‐fat milk for 2h at 25°C and incubated with primary antibodies, including USF1, E‐cadherin, vimentin, PCNA, Bax and GAPDH, respectively, the PVDF membranes were eventually incubated with secondary antibody (Cell Signaling Technology).

### 2.11 Cell immunofluorescence

After CCLP1 cell was washed by PBS for 3 times, cells were fixed in 4% formaldehyde for 10 minutes. Then, cells were permeabilized with 0.5% Tx‐100 in PBS for 5 minutes, blocked in 10% FBS and incubated with appropriated primary antibodies for 1 hour at indoor temperature. After several washes, samples were incubated with secondary antibodies for 1 hour at indoor temperature. Images were acquired using Fluorescence Inversion Microscope System (Leica).

### 2.12 Statistical analyses

Each experiment was performed for at least three times. Unless otherwise noted, data were presented as the mean ± SD and analysed by Student's *t* test using the SPSS software 19.0 package (IBM) or GraphPad Prism 5.01 (GraphPad Software, Inc). The correlations between ZFAS1 and miR‐296‐5p as well as USF1 in human tissues were analysed by Spearman's rank test. Overall survival analysis was evaluated by the Kaplan‐Meier curve and assessed using the log‐rank test. *P* < .05 was statistically significant.

## 3 RESULTS

### 3.1 Up‐regulated ZFAS1 transcription in CCA tissues is associated with patients’ clinicopathological parameters and indicates an undesirable overall survival

**Figure 1:**
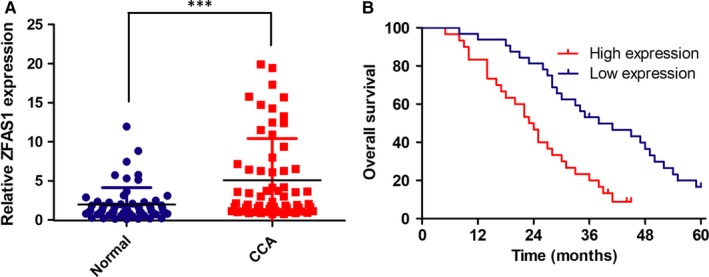
ZFAS1 was up‐regulated in cholangiocarcinoma (CCA) and indicated a poor survival. A, ZFAS1 was relatively up‐regulated in tumour tissues than adjacent normal tissues. B, Relatively high ZFAS1 expression group is associated with worse patient survival (OS)

To investigate the transcription level of ZFAS1 in paired CCA and adjacent normal tissues, quantitative real‐time PCR was carried out to quantify ZFAS1 expression. The results showed that ZFAS1 was significantly overexpressed in CCA tissues with *P*‐value < .05 in Figure 1A, and the expression level of ZFAS1 was 2.25‐fold higher in the CCA samples than the paired normal tissues. And then, 64 patients were categorized into low (< the average value) and high (> the average value) groups according to the average expression level of the ZFAS1. As shown in Table 2, all data were calculated by Fisher's exact tests which indicated that the up‐regulation of ZFAS1 was analysed to be associated with lymph node invasion (*P* = .042), TNM stage (*P* = .023) and postoperative recurrence (*P* = .035). However, no significance was found between ZFAS1 expression and other parameters. Kaplan‐Meier analyses and log‐rank tests were performed to assess the relation between aberrant expression and overall survival (OS). Figure 1B indicated that relative high expressional ZFAS1 was relevant to worse overall survival.

**Table 2:** Association between ZFAS1 expression and clinicopathological features

Features	No. of patients	ZFAS1 expression		*P*‐value
	64	High	Low	
Gender				
Male	30	17	13	.209
Female	34	13	21	
Age				
<60	35	18	17	.460
≥60	29	12	17	
Vascular invasion				
Positive	20	11	9	.427
Negative	44	19	25	
Tumour location				
Intrahepatic	13	4	9	.261
Perihilar	24	14	10	
Distal	27	12	15	
Lymph node invasion				
Present	27	17	10	.042
Absent	37	13	24	
HBV infection				
Positive	29	13	16	.806
Negative	35	17	18	
Differentiation				
Well/Moderate	23	10	13	.796
Poor	41	20	21	
TNM stage				
I‐II	36	12	24	.023
III‐IV	28	18	10	
Postoperative recurrence				
Present	42	24	18	.035
Absent	22	6	16	
Serum CA19‐9				
>37 µ/mL	39	22	17	.074
≤37 µ/mL	25	8	17	

Abbreviations: CA19‐9, carbohydrate antigen 19‐9; CEA, carcinoembryonic antigen; HBV, hepatitis B virus; TNM stage, tumour‐node‐metastasis stage.

### 3.2 ZFAS1 is overexpressed in CCA cell lines and knockdown of ZFAS1 inhibits cell proliferation migration and invasion in vitro and in vivo

**Figure 2:**
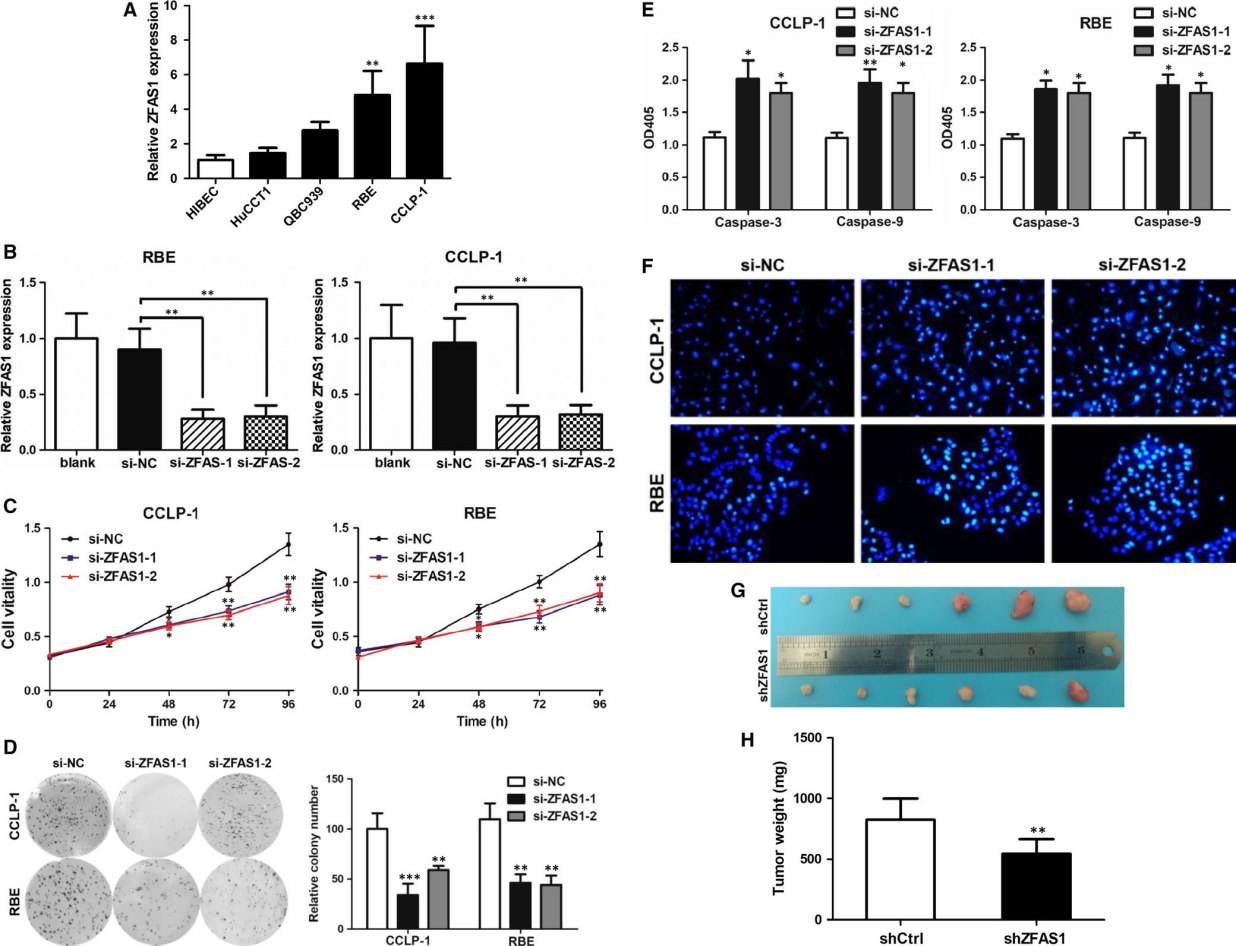
ZFAS1 was overexpressed in CCA cells and knockdown of ZFAS1 significantly reduced cell proliferation vitality and promoted cell apoptosis. A, ZFAS1 was significantly up‐regulated in RBE and CCLP1 cells compared with HIBEC. B, The si‐ZFAS1‐1 and si‐ZFAS1‐2 suppressed ZFAS1 expression in RBE and CCLP1. C, Knockdown of ZFAS1 suppressed RBE and CCLP1 cell vitalities. D, Knockdown of ZFAS1 suppressed RBE and CCLP1 colony formation. E, Knockdown of ZFAS1 promoted RBE and CCLP1 apoptosis detected by caspase 3 and 9. F, Knockdown of ZFAS1 promoted RBE and CCLP1 apoptosis detected by Hoechst 33342 staining. G, Silence of ZFAS1 suppressed tumour growth in vivo. H, Silence of ZFAS1 suppressed tumour weights

To investigate the role of ZFAS1 in cell lines, four types of CCA cells and one negative control were used and qRT‐PCR was performed, and the data demonstrated that ZFAS1 expression in CCLP‐1 and RBE cells was relatively higher compared with the negative control of HIBEC cell as shown in Figure 2A. Known that ZFAS1 was up‐regulated in both CCA tissues and cell lines, it became a requisite point to determine whether the effects of ZFAS1 silencing could inhibit tumour cell growth activity. Two siRNAs (si‐ZFAS1‐1 and si‐ZFAS1‐2) targeting ZFAS1 were transfected into the previously screened out CCLP‐1 and RBE cells, and qRT‐PCR was carried out for the evaluation. The data indicated that both of the two selected siRNAs could significantly decrease ZFAS1 expression as shown in Figure 2B. In that case, CCK‐8 and colony formation assays were performed and the results indicated that compared with siRNA of negative control (si‐NC), silence of ZFAS1 significantly suppressed cell growth (Figure 2C), and the tumour cell colonies formed troublesomely for the very inhibition of ZFAS1 (Figure 2D). Interestingly, the activities of caspase‐3 and caspase‐9 were both higher in the ZFAS1 silence group than the negative control (Figure 2E) and the Hoechst 33342 staining outcomes also presented severer apoptosis of cells resulted from ZFAS1 inhibition (Figure 2F). To further investigate whether ZFAS1 expression could have impact on tumorigenesis in vivo, BALB/c nude mice (n = 10) were injected with transfected CCLP‐1 cells (shZFAS1/ shCtrl). In accordance with previous in vitro outcomes, tumour growth of shZFAS1‐transfected cell injection group showed a relative slower progress on tumour formation (Figure 2G). Furthermore, tumour weights examination after inoculation for 18 days displayed that the weights of shZFAS1 transfected injection tumours were significantly lower than shCtrl group (Figure 2H).

### 3.3 Knockdown of ZFAS1 inhibits cell migration and invasion by reversing epithelial‐mesenchymal transition

**Figure 3:**
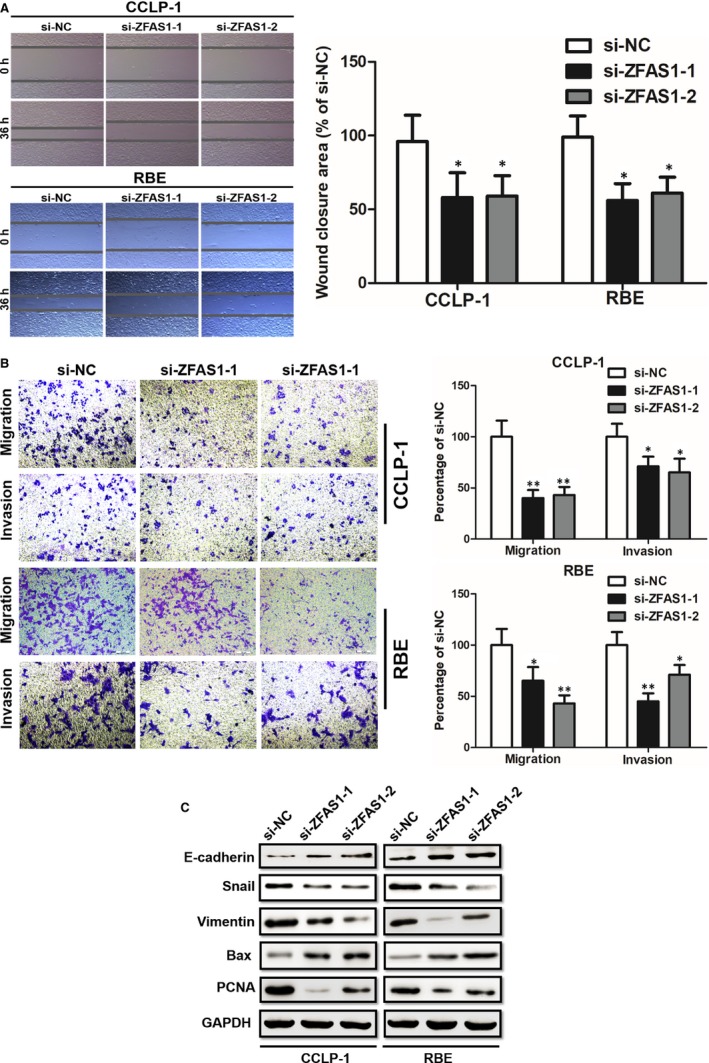
Knockdown of ZFAS1 suppressed RBE and CCLP1 cells metastasis. A, Knockdown of ZFAS1 suppressed RBE and CCLP1 cells migration detected by wound healing assay. B, Knockdown of ZFAS1 suppressed RBE and CCLP1 cells migration detected by migration and invasion. C, The si‐ZFAS1‐1 and si‐ZFAS1‐2 transfection promoted cells E‐cadherin and Bax expression but suppressed Snail, vimentin and PCNA expression

Given that ZFAS1 was related with lymph node invasion, we further explored the potential impact of ZFAS1 on migration and invasion by performing wound healing assays and transwell assays. Compared with si‐NC, cells transfected either with si‐ZFAS1‐1 and si‐ZFAS1‐2 presented a dramatically weaker migration and invasion ability (Figure 3A and B). Since epithelial‐mesenchymal transition (EMT) has already been proved to be one crucial mechanism correlated with cell metastasis capability and protein PCNA, Bax are associated with cell proliferation and apoptosis regulation, respectively. We then conducted the Western blot analyses to assess whether ZFAS1 could be involved into EMT and proliferation progression. After transfected with si‐ZFAS1‐1, si‐ZFAS1‐2 or si‐NC, we found E‐cadherin and Bax were increased but the expression levels of others were decreased (Figure 3C).

### 3.4 Knockdown of ZFAS1 Increased the Expression of miR‐296‐5p and Decreased the Expression of USF1

**Figure 4:**
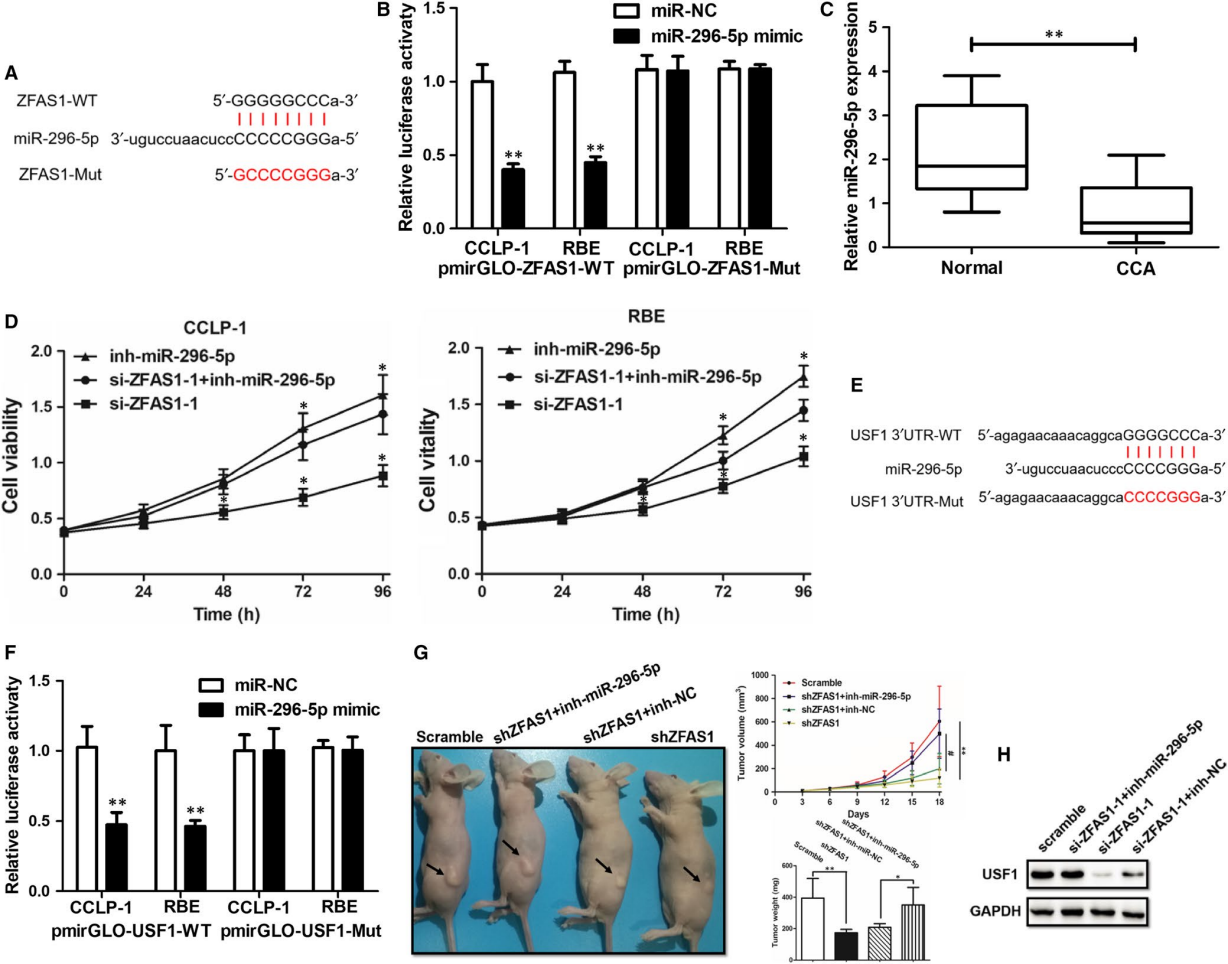
ZFAS1 regulated USF1 expression by directly binding miR‐296‐5p as a ceRNA manner and affected tumour growth in vivo. A, The binding motif of miR‐296‐5p on ZFAS1 3′UTR predicted by TargetScan database. B, Relative luciferase activity between miR‐296‐5p and ZFAS1 was pulled up by shifting sequence using pmirGLO‐ZFAS1‐Mut. C, The miR‐296‐5p was significantly down‐regulated in tumour tissues compared with adjacent normal tissues. D, Inhibition of miR‐296‐5p rescued the si‐ZFAS1‐1 transfected RBE and CCLP1 cell vitality. E, The predicted motif and shifted sequences of USF1 3′UTR. F, Relative luciferase activity between miR‐296‐5p and USF1 3′UTR was pulled up by shifting sequence using pmirGLO‐USF1‐Mut. G, Inhibition of miR‐296‐5p rescued the shZFAS1 transfected xenograft tumour growth on volumes and weights in vivo. H, Up‐regulated ZFAS1 or inhibited miR‐296‐5p promoted USF1 expression

Thanks to the help of computer‐aided algorithms, the binging motif sequences were predicted as Figure 4A. Wild and mutant ZFAS1 sequences were inserted into pmirGLO reporter, and the luciferase activity in CCLP‐1 and RBE cells were cotransfected with miR‐296‐5p mimics or miR‐NC and pmirGLO‐ZFAS1‐WT or pmirGLO‐ZFAS1‐Mut. Luciferase activities were normalized to renilla luciferase (Figure 4B). Came back to examine the expression of miR‐296‐5p in clinical tissues, the qRT‐PCR revealed that miR‐296‐5p significantly down‐regulated in CCA tissues (Figure 4C). And the inhibition of miR‐296‐5p could promote cell growth which was tested by CCK‐8 (Figure 4D). And the binding motif of miR‐296‐5p and USF1 3′UTR was predicted by TargetScan database (Figure 4E). The luciferase activity in CCLP‐1 and RBE cells was evaluated again by cotransfected with miR‐296‐5p mimics or miR‐NC and pmirGLO‐USF1‐WT or pmirGLO‐USF1‐Mut. The outcomes indicated transfection with miR‐296‐5p mimics inhibited the intensity of the luciferase reporter containing USF1 wild type relative to the miR‐NC (Figure 4F). Next step, shZFAS1, si‐ZFAS1‐1, miR‐296‐5p inhibitor (inh‐miR‐296‐5p) and related negative controls were conducted in vivo (Figure 4G) which reflected the tumour‐promoting role in both growing speed and tumour weight. And Western blot with CCLP‐1 cell (Figure 4H) demonstrated the evidence of ZFAS1/miR‐296‐5p/USF1 axis. So that, knockdown of ZFAS1 decreased miR‐296‐5p but increased USF1, and either of ZFAS1 and miR‐296‐5p or miR‐296‐5p and USF1 3′UTR could bind directly.

### 3.5 USF1 is up‐regulated in CCA cells and knockdown of USF1 inhibits cell proliferation and migration

**Figure 5:**
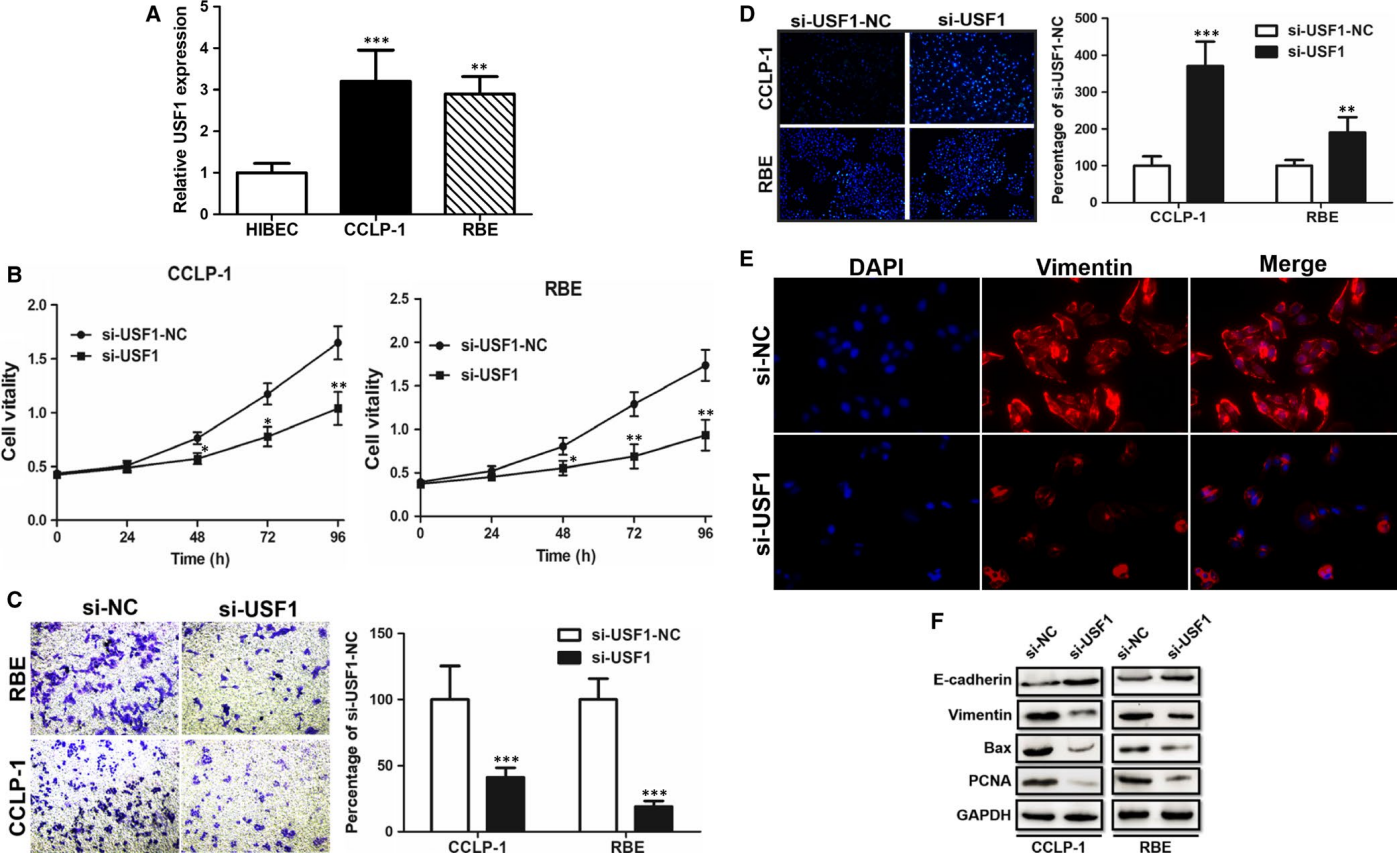
USF1 was up‐regulated in RBE and CCLP1 cells and promoted cell proliferation and metastasis. A, USF1 was up‐regulated in RBE and CCLP1 cells compared with HIBEC. B, Knockdown of USF1 significantly suppressed RBE and CCLP1 cell vitalities evaluated by CCK‐8 assay. C, Knockdown of USF1 suppressed RBE and CCLP1 cell migration ability. D, Knockdown of USF1 promoted RBE and CCLP1 apoptosis detected by Hoechst 33342 staining. E, Knockdown of USF1 suppressed cell EMT progression detected by immunofluorescence of vimentin expression and cell morphology. F, Knockdown of USF1 suppressed Bax and PCNA expression but enhanced E‐cadherin expression

The qRT‐PCR was performed to test the expression of ZFAS1 in cells. The siRNA to silence USF1 (si‐USF1) and negative control (si‐USF1‐NC) were formed and found USF1 was down‐regulated in CCLP‐1 and RBE cells (Figure 5A). Again, we performed CCK‐8 assay, transwell assay to validate the role of USF1. The outcomes indicted that si‐USF1 group induced a relative undesirable proliferation (Figure 5B) and metastasis (Figure 5C). The Hoechst 33342 staining also showed the same trend that si‐USF1 transfected leaded to a severe cell apoptosis (Figure 5D). The cell immunofluorescence results displayed that the expression of vimentin turned down after transfection with siRNA. Knockdown of USF1 suppressed cell EMT progression detected by immunofluorescence of vimentin expression and cell morphology (Figure 5E). The Western blot evaluated that despite E‐cadherin, PCNA, Bax and vimentin were suppressed as a result of USF1 silence (Figure 5F).

### 3.6 USF1 induces CCA‐related transcription by the prediction of binding to the promoter regions

**Figure 6:**
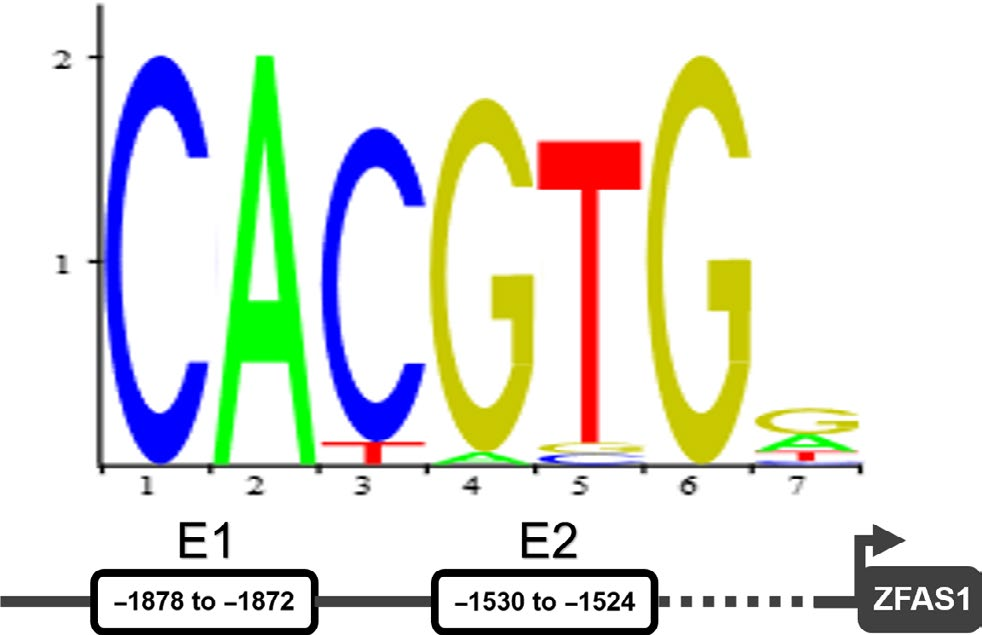
The prediction of the binding locations between USF1 and the promoter of ZFAS1

Plentiful evidence has proved that transcription factors such as SP1 and USF1 could activate the transcription of targets by binding to specific region of the very promoter including lncRNAs. Although we have found ZFAS1 was overexpressed in CCA tissues and cell lines, the factors involved in ZFAS1 dysregulation remained unknown. In the present study, we predicted that the potential transcription factor USF1 could bind to ZFAS1 promoter region at two binding sites E1 (−1878 to −1872) and E2 (−1530 to −1524) with relative high scores by using JASPAR database (Figure 6). After that, we assessed the binding motifs of several recently discovered CCA‐related lncRNAs’ promoters by JASPAR and found USF1 might bind some lncRNAs’ promoters with relative high scores (Table 3) to activate oncogenic lncRNAs.

**Table 3:** Prediction of motifs by JASPAR

LncRNA	Predicted site sequence	Score (max)
ZFAS1	CACATGG	7.811
SOX2‐OT	CATGTGT	7.096
AFAP1‐AS1	CACGTGG	11.491
SNHG1	CATGTGG	8.367
TP73‐AS1	CACGTGT	10.220
CRNDE	CAGGTGG	7.068
CCAT1	CACATGG	7.811

## 4 DISCUSSION

Cholangiocarcinoma, also known as biliary tract cancer, is recognized as one of the most malignancies among all digestive system tumours for years. Currently, various treatments have been attempted to cure this disease, including radiotherapy, chemotherapy, biotherapy and others, but radical resection still has been proved to be the key approach to fight this cancer. In that case, finding new ways of therapies and preventive strategies against CCA have become particularly important. Thanks to the development of molecular research and laboratory technique, plentiful types of biological molecules have been screened out to have specific functions during CCA tumorgenesis and gradual progress.

LncRNAs are one huge class of those newly focused specific molecules to have crucial impact on CCA, and their functional modes were relatively unique to the classic protein‐coding factors, which could regulate gene transcription and post‐transcription without coding proteins. Some of the CCA‐related lncRNAs were excavated to play vital roles during the genesis and development of CCA recent years, for instance lncRNA TUG1 suppression inhibits CCA metastasis potential by reversing EMT, SNHG1 binds to the histone methyltransferase enhancer of EZH2 and PRC2 to alter the CCA migration as well as proliferation in vitro and in vivo, AFAP1‐AS1 promotes the CCA proliferation and metastasis while providing potential therapeutic target for CCA.^29^, ^30^, ^31^, ^32^

LncRNA ZFAS1, antisense to the 5’ end of ZNFX1 promoter, has been demonstrated aberrantly expressed in many cancers and equipped with different functional mechanisms, which we have previously described. As one of our previous works shows that a larger number of studies have indicated that the ZFAS1 is overexpressed in many human cancers, such as breast cancer, gastric cancer, colorectal cancer hepatocellular cancer and many others, which is one important evidence to imply the potential role of ZFAS1 in the progression of human tumours.^33^ So that, we performed a series of experiments to detect whether ZFAS1 could act as one important factor in CCA.

In the present study, ZFAS1 was firstly found to have a similar expression trend as in other tumours that ZFAS1 was up‐regulated in CCA human tissues compared to the adjacent normal tissues. The evaluation between relative ZFAS1 expression and clinicopathological features of 64 patients with no chemoradiotherapy history indicated that this factor could signicantly correlated with some of the patients' parameters. The data displayed that ZFAS1 expression was associated with lymph node invasion, TNM stage and postoperative recurrence, which might be one initial evidence to display the correlation between ZFAS1 and CCA. The Kaplan‐Meier curves provided that patients with relative low ZFAS1 expression enjoyed a better overall survival. After that, the effects of up‐regulated ZFAS1 were detected by CCA cells that preserved in our laboratory within 6 months. We found that ZFAS1 was up‐regulated in most of our preserved CCA cells, and after ZFAS1 silencing in CCLP‐1 and RBE cells, the CCK‐8 assay, colony formation, migration and invasion abilities all significantly glided downside but apoptosis by Hoechst 33342 and caspase‐3. EMT, a major mechanism involved in tumour metastasis, causes the loss of cell‐cell adhesion and increases migration and invasion capabilities. We further detected that the knockdown of ZFAS1 could regulate EMT alteration. Meanwhile, the PCNA and Bax were also explored, and the outcomes indicated that ZFAS1 is one lncRNA that might impact CCA growth and metastasis by alteration of EMT, PCNA and Bax.

After validating the role of ZFAS1 as an oncogene in CCA, the underlying mechanisms of tumour malignant behaviours aroused our interest. LncRNAs have been proved to have the abilities of modulating gene expression via multiple mechanisms, and competitively binding to microRNAs is the most crucial approach to conduct its function. Like most researcher presented, we performed a series of bioinformatics via online computer‐aided algorithms and screened out several potentially combined partner microRNAs. Consequently, detecting whether miR‐296‐5p could be directly targeted by ZFAS1 became our concern. Online computer‐aided algorithms were conducted and predicted the target binding sites and the luciferase assay verified the prediction. After evaluation with cell lines, miR‐296‐5p was finally selected. Interestingly, miR‐296‐5p was tested to be relatively down‐regulated in the paired tissues from the same 64 patients, which as a result inspired our confidence to proceed. TargetScan was used for further prediction and found USF1 as one potential downstream target of miR‐296‐5p. In that case, we conducted experiments not only in vitro but also in vivo and found ZFAS1/miR‐296‐5p/USF1 regulation might work as one approach to influence tumour progression.

USF1 has been evaluated as transcription factor that could bind gene‐specific binding motif in the previous research and activated gene expression.^34^ Consequentially, we attempted with the help of JASPAR and discovered that USF1 might be an important transcription factor for ZFAS1. In addition to that results, the associative property of USF1 on the motifs of CCA‐related lncRNAs was also evaluated. The currently found results indicated ZFAS1 as one oncogenic lncRNA might function in the way of ZFAS1/miR‐296‐5p/USF1, and the feedback of USF1 might continuously activate oncogenic lncRNAs’ transcription.

In conclusion, the results presented that overexpressed ZFAS1 is associated with dismal prognosis for cholangiocarcinoma patients and this aberrant modulates proliferation and metastasis via miR‐296‐5p/USF1, which might activate oncogenes expression.

## 5 AUTHOR CONTRIBUTION

Study idea, design and manuscript preparation: Zhenglong Li, Xingming Jiang. Data collection and interpretation: Lining Huang, Jinglin Li and Daolin Ji. Experiment performance and data analysis: Yi Xu, Zhenglong Li and Kaiming Leng. Final correction and review: Yunfu Cui.

## CONFLICT OF INTEREST

The authors confirm that there are no conflicts of interest.
